# Heterozygous Mutation of Sodium Voltage-Gated Channel Alpha Subunit 2 and Pumilio Homolog 1 Genes in a Pediatric Patient: A Case Report

**DOI:** 10.7759/cureus.30577

**Published:** 2022-10-22

**Authors:** Fawzia Al-Sharif, Feryal O Attiah, Nada A AlKhateeb, Hussein o Taher, Mohammed F Alamer, Raneem y Gazzaz, Muayad o Ba Sahel, Rena M Alsharif

**Affiliations:** 1 Pediatrics, Saudi German Hospital Jeddah, Jeddah, SAU; 2 Medicine, King Abdulaziz University Hospital, Jeddah, SAU; 3 Medicine, Batterjee Medical College, Jeddah, SAU; 4 Medicine, College of Medicine, Umm Al-Qura University, Mecca, SAU

**Keywords:** seizures, scn2, epilepsy, pum1, rna-binding protein

## Abstract

Epilepsy is a neurological condition brought on by recurrent and spontaneous seizures in patients with hypersynchronous neuronal ensemble activity. These spontaneous seizures appear to be brought on by increased neuronal excitability and synaptic synchronization. The development of neuronal hyperexcitability and acquiring epilepsy is still poorly understood. Cell differentiation and development might be related to the pumilio RNA-binding family member 1 (Pumilio 1 (*PUM1*)). Complete deficiency of this gene causes misregulation of the proteins involved in the control of neuronal excitability. Furthermore, the voltage-gated sodium channels alpha subunit 2 (*SCN2A*) triggers action potentials in brain neurons, and a variety of severe hereditary epilepsy syndromes are caused by their mutation. Here, we present a rare case of a seven-year-old female with co-occurrence of two genetic mutations in the pumilio homolog 1 (*PUM1*) and sodium voltage-gated channel alpha subunit 2 (*SCN2A*).

## Introduction

The sodium voltage-gated channel alpha subunit 2 (*SCN2A*) gene is necessary for regulating the onset and spread of action potentials in neurons and muscles. The *SCN2A* gene might develop a mutation that results in a gain of sodium voltage 1.2 (Nav1.2) function, which is thought to cause early-onset epilepsy. This mutation has also been strongly linked to several neurodevelopmental disorders, including forms of epileptic disorders such as infantile epileptic encephalopathy, benign familial infantile seizures, autism spectrum disorders (ASD), intellectual disability, and schizophrenia [1].

Status epilepticus is a continuous seizure activity or recurrent seizure activity without regaining consciousness lasting for five minutes or more [2]. Depending on the type and duration of seizures, status epilepticus can have long-term effects such as neuronal death, neuronal injury, and change in neural networks [3]. Status epilepticus may be convulsive, non-convulsive, focal motor, or myoclonic; any can become refractory and super-refractory as well, requiring intensive care unit (ICU) admission [2].

The Pumilio homolog 1 (*PUM1*) gene is a member of the Pumilio and fem-3 binding factor (PUF) family of evolutionarily conserved RNA-binding proteins. The translation of this gene gives a functional protein that has been preserved throughout evolution in both lower vertebrates and invertebrates; this human protein may be involved in the translational control of embryogenesis and cell development and differentiation [4]. Mutations in *PUM1* can lead to spinocerebellar ataxia 47 (SCA47) [5].

## Case presentation

We present here a seven-year-old female who was initially seen for an upper respiratory tract infection (URTI) (cough and runny nose) at the age of eight months when she was diagnosed with delayed developmental milestones for her age. She was on citicoline 7 drops, piracetam 3 mL, folic acid 5 mg, calcium carbonate 3 mL, cerebrolysin 1 mL three times weekly, and oral iron 3 mL. She was born at term via C-section to non-consanguineous parents, with a birth weight of 2,800 g without dysmorphic features, and not requiring neonatal ICU. Thyroid screening was normal. She had normal development until the age of three months when she started showing delays in the gross motor parameters of developmental milestones. History declared that she had a squint and shaking of eyes, with retinal and optic nerve affection. She started recognizing her mother at three weeks and developed a social smile at two months.

Upon examination, she had no dysmorphic features and was vitally stable. Her length was at the 10th centile (65 cm), her weight was below the fifth centile (6.3 kg), and her head circumference was at the 10th centile (42 cm). She was slightly hypotonic and had a right plantar extensor reflex. Eye examination showed nystagmus on looking to the left. She cannot fix her vision but can look at the light. Magnetic resonance imaging (MRI) of the brain was unremarkable. Developmentally, she can follow objects, has good movements of both hands and feet, cannot sit or support her neck, and sometimes can hold things with her hands.

Biochemically, the thyroid function, serum lactic acid, random blood sugar, and venous blood gas tests were normal. A preliminary assessment revealed an elevation of very long-chain fatty acids (VLCFA) shown in Table 1. The plasma VLCFA analysis is used frequently as a diagnostic test for adrenoleukodystrophy.

**Table 1:** Results of VLCFA in serum The assay depends on the demonstration of increased levels of C26:0 and increases in the C26:0/C22:0 and C24:0/C22:0 ratios. The number after the “C” is the number of carbon atoms in the chain. VLCFA: very long-chain fatty acids

	Patient result	Normal range	Interpretation
C22:0	27.700 mg/L	1.827-22.161 mg/L	High
C24:0	20.200 mg/L	1.228-18.837 mg/L	High
C26:0	0.229 mg/L	<1.030 mg/L	Normal
C24/C22 ratio	0.729	0.665-1.008	Normal
C26/C22 ratio	0.008	0.009-0.069	Low

The patient was planned for abdominal ultrasonography to access suprarenal abnormalities, phytanic acid level, and patellar X-ray to rule out any peroxisomal disorder, which revealed no abnormalities. She started 12 sessions of a physiotherapy course.

By the age of 13 months, she was able to sit with support and roll in bed. All metabolic laboratory tests were negative. Brain electrical activity mapping was performed and showed a focal right temporal epileptiform discharge with some left-sided propagation. MRI of the brain showed no abnormality. The skyline view of the patella revealed unremarkable osseous structures for her age. She needed extensive physiotherapy daily for three months.

The following medications were added to her drug regimen: omega 3 4 mL, vitamin E 400 ug, vitamin B12 one ampule per week, and coenzyme Q10 three times per week. At 16 months of age, the patient was improving on physiotherapy as she started to sit unsupported for a few minutes, and she was able to support her neck. An X-ray of the hip was done, and developmental dysplasia was ruled out.

At the age of 2.5 years, she was found to have weakness in the lower limbs more than in the upper limbs and was able to feed on a soft diet orally. Laboratory results showed moderately elevated ammonia and mildly elevated long-chain fatty acid.

At the age of two years and eight months, she developed her first seizure. She presented to the emergency room (ER) with vomiting and decreased activity level but no diarrhea or fever. In the ER, she developed her first generalized tonic-clonic seizure lasting for 20 seconds with uprolling of the eyes, no desaturation, and preserved sphincteric function. She was vitally unstable with a heart rate (HR) of 156 beats per minute, respiratory rate (RR) of 30 breaths per minute, and blood pressure (BP) of 91/59 mmHg. She was afebrile, had an oxygen saturation of 92%, and reduced level of consciousness with a Glasgow Coma Scale (GCS) score of 7/15. She was hypotonic and had decreased reflexes, reduced skin turgor, and sunken eyes. Her pupils were equal, round, and reactive to light and accommodation, and she had no neck rigidity. Her chest had decreased air entry with crackles on the right side. She was admitted and given electroconvulsive therapy (ECT). Electroconvulsive therapy is useful to decrease seizure duration and increase seizure threshold, making it one of the choices as adjunctive therapy in some cases of epilepsy.

Blood works were significant for low hemoglobin, low hematocrit, elevated erythrocyte sedimentation rate (ESR), low serum creatinine, low albumin, and elevated D-dimer as shown in Table 2. Arterial blood gas showed a picture of severe respiratory acidosis with severely decreased PH, elevated PCO2, and decreased HCO3 as demonstrated in Table 3. Her chest X-ray showed right-sided lung opacity with pleural effusion, and for that, she received ceftriaxone and managed as a case of lower respiratory tract infection.

**Table 2:** Laboratory test results ESR: erythrocyte sedimentation rate; FEU: fibrinogen equivalent unit

Test	Patient result	Reference range	
Hemoglobin	10.7 g/dL	11-14 g/dL	
Hematocrit	32.1%		34%-40%
ESR	30 mm/hour		6-12 mm/hour
Albumin	3.1 g/dL		3.4-5 g/dL
Creatinine	0.20 mg/dL		15-38.5 mg/dL
D-dimer	0.95 mg/L FEU		Up to 0.55 mg/L FEU

**Table 3:** Arterial blood gas results ABG: arterial blood gas; PCO2: partial pressure of carbon dioxide; HCO3: bicarbonate

Test		Patient result	Reference range
ABG	PH	7.2	7.35-7.45
	PCO2	50.9 mmHg	35-45 mmHg
	HCO3	19.8 mmol/L	22-26 mmol/L

Two months later, she presented again to the ER with noisy breathing for one hour while sleeping. She later developed focal seizures in the form of uprolling in the eyes, staring in space, and tonic extension of the leg. That was her second noted event, which was managed with midazolam, oxygen, and admission to be under observation. She remained vitally stable. Her father refused admission due to financial issues, and she was discharged on Keppra 2.2 mL twice daily.

At four and a half years, the patient had an episode of fever followed by repeated convulsions, excessive salivation, and uprolling of eyeballs lasting for a few minutes. She was managed at home only, and treatment was given on an outpatient department (OPD) basis as a case of upper respiratory tract infection (URTI). On the next day, she developed absence seizures lasting 90 minutes as per her parents. On examination, the patient was found to have a decreased level of consciousness, cyanosis, unresponsiveness to stimuli, apnea, and frothy secretions from the mouth, in addition to attacks of focal convulsions on the right side of the body. During the assessment, the patient developed bradypnea, a code blue was announced at the outpatient department, and the resuscitation team resuscitated and intubated the patient. She was admitted to the pediatrics intensive care unit (PICU) for further management. Upon admission, her C-reactive protein (CRP) was high at 28.72 mg/L. The patient stayed in the PICU for one day; she was vitally stable and on an oxygen mask. She was discharged the next day.

At five and a half years of age, the patient was seen in the pediatrics outpatient department. She was hypotonic, with no dysmorphic features or abnormal movement. A systemic examination of her chest and heart was normal. The patient still cannot form speech, communicate, or walk. Electroencephalogram (EEG) showed mild diffuse non-specific disturbances of electrical cortical rhythms. No epileptiform discharges were noted during this EEG. She also complained of chronic constipation as she opens her bowel every three days. Six months later, she developed a focal seizure of the left side of her body, which lasted for 10 minutes, and for that, the Keppra dose was increased to 3 mL twice daily.

At the age of seven years, whole exome sequencing (WES) using an Agilent SureSelect V6 kit (Agilent, Santa Clara, CA, USA) was performed, and two gene mutations were recognized: heterozygous variants in the sodium voltage-gated channel alpha subunit 2 (*SCN2A*) and Pumilio homolog 1 (*PUM1*) genes. The *SCN2A* gene is responsible for developmental delay and epileptic encephalopathy, while the *PUM1* gene is responsible for spinocerebellar ataxia.

On her last visit, she is still unable to walk, communicate, or speak. She has head support, sits with assistance, has poor hand function and weak hand-to-mouth movement, can hold things for a minimal time, has social anxiety, and can recognize relatives. Throughout her hospital course over the years, she had multiple episodes of otitis media and upper respiratory tract infection, which were managed accordingly.

## Discussion

Mutations causing loss of function (LOF) or changes in the *SCN2A* structure protein could disturb the sodium channel function. This would establish a loss or decrease in the excitability of neuronal cells, which could be a cause of autism spectrum disorder or could attain hyperexcitability of neuronal cells [6]. Neuronal cell hyperexcitability is denoted as infantile seizures. Loss-of-function (LOF) mutation, which does not involve hyperexcitable neurons, can be one of the possible causes of severe phenotypes with seizures. Later on, during development, decreased excitability of unmyelinated cortical inhibitory neurons would lead to cortical network hyperexcitability [6]. It has been suggested that through *SCN2A* mutations, the ones causing a gain of function (GOF) and increased neural excitability result in epilepsy. The associated phenotype varieties with different types of *SCN2A* mutations will provide a chance to investigate more about the relationship between phenotype and channel function [7]. The cause behind epilepsy plays a major role in determining the clinical course and prognosis. Epilepsy could broadly be classified according to its etiology into six different groups: metabolic, structural, infectious, genetic, immune, and idiopathic [8].

Genetic testing is widely practiced most commonly in pediatric neurology. Almost half of the children with epilepsy have a genetic cause. At present, *SCN1A*, *SCN2A*, *SCN3A*, and *SCN8A* are voltage-gated sodium channel genes stated to be one of the causative genes of epilepsy. Among the mentioned genes, *SCN1A* is the most common causative gene of epilepsy. *SCN2A* is the second most common gene causing epilepsy [9]. Epilepsy caused by *SCN2A* variants most commonly starts in early childhood and has a vast phenotypic spectrum, ranging from self-limited epilepsy owning a good prognosis to epileptic and developmental encephalopathy. Most patients with *SCN2A* variants respond well to sodium channel blockers [8].

A loss-of-function mutation of *PUM1* has shown an increase in the ataxin 1 (ATXN1) mRNA level, leading to the accumulation of ATXN1 in Purkinje cells, which are located in the cerebellar cortex of the brain [10]. This accumulation leads to motor dysfunction, resulting in spinocerebellar ataxia type 1 [11]. Recently, it has been suggested that the *PUM1* gene causes developmental disorders, specifically Pumilio 1-associated developmental disability, ataxia, and seizure syndrome (PADDAS) [12]. This supports the evidence that other brain areas located near the cerebellar cortex could be affected by *PUM1* mutation.

Whole exome sequencing (WES) has been traditionally defined as the encompassing sequence of all exons of protein-coding genes in the genome. It has been shown that WES covers between 1% and 2% of the genome, which depends on its different species [13]. WES added much to the clinical practice impressively, because it can cover actionable genome areas that determine the variations in the regions of the exon, detecting variants of mutations causing the diseases [14]. Most WES technical tools are based on the formation of a variant call format (VCF) file from raw sequencing data [15]. There is substantial evidence that using WES in clinical practice can enhance genetic disease diagnosis and aid in the treatment plan, which may enhance patient health outcomes and enable more effective utilization of healthcare resources [16]. Despite making up just around 1% of the whole human genome, the coding region causes up to 85% of mutations known to cause Mendelian disorders [17]. After performing WES for our patient, the result summary is listed in Table 4.

**Table 4:** Whole exome sequencing result The whole exome sequencing performed for the patient identified heterozygous variants in the *SCN2A* and *PUM1* genes. DNA: deoxyribonucleic acid; CADD score: Combined Annotation Dependent Depletion score

Gene	DNA mutation	Zygosity	CADD score
SCN2A	c.4595T>C	Heterozygous	19.18
PUM1	c.2722-19A>T	Heterozygous	15.41

Levetiracetam is a newly developed antiepileptic drug, a product of piracetam, that has been shown to have good pharmacokinetics and almost 100% bioavailability. Levetiracetam reaches the peak plasma concentration level about an hour after oral administration. It is clinically used in treating various states of epilepsy, commonly convulsive epilepsy in pediatric patients [18]. It has a unique site in the brain, which is the synaptic vesicle protein (SV2A), to act on achieving its antiepileptic effect. Levetiracetam regulates the function of SV2A by decreasing the vesicle release rate [19]. Intravenous levetiracetam can be given to patients who cannot tolerate oral levetiracetam, and its’ peak plasma concentration is reached in only 5-15 minutes.

Physiotherapy plays a major role in the recovery of epilepsy patients, especially among the pediatric group [20], as physiotherapy induces physiological and psychological benefits for such patients. It is strongly recommended for patients with diseases or accidents that impair their ability to move or coordinate [21]. While the majority of epilepsy patients may not require physical therapy (PT), individuals who are disabled or have restricted mobility may be good candidates, such as our patients. In these situations, physiotherapy can improve mobility and coordination using a variety of strategies, such as skill development, exercise, and stretching [22].

Physical therapy should be used as supplemental therapy for patients with epilepsy not only to manage seizures but also to improve mental health [23]. Support from health authorities, social workers, and sports coaches and initiatives to educate and encourage patients to be more active are required to achieve these aims. Some physiotherapy goals are to become strong enough to hold patients’ heads, to be able to maintain their trunk at midline with balance and motor control, to engage a variety of muscle groups, to support their extremities’ and trunks’ basic motion, to recognize patients’ strengths, to concentrate on what the patient can accomplish rather than what they can’t, and to raise awareness among patients [24].

## Conclusions

Treating pediatric patients could sometimes be challenging, especially for patients diagnosed with rare genetic mutations. As these genetic mutations still have no definitive treatment, many medications and enzyme replacement therapy are still under clinical trials. We report the first case of a heterogeneous mutation in both *SCN2A* and *PUM1* genes occurring in a pediatric patient. We want to emphasize how essential physiotherapy is for patients with neurological disorders such as epilepsy. In addition, it is important to provide fall prevention programs and exercise programs as it is recommended, which will improve fitness and aid in reducing seizures from reoccurring. We desire further studies to be conducted in the near future providing definitive treatment for such patients to improve their quality of life.
